# Transcription Factor Runx3 Is Induced by Influenza A Virus and Double-Strand RNA and Mediates Airway Epithelial Cell Apoptosis

**DOI:** 10.1038/srep17916

**Published:** 2015-12-08

**Authors:** Huachen Gan, Qin Hao, Steven Idell, Hua Tang

**Affiliations:** 1 Department of Cellular and Molecular Biology, The University of Texas Health Science Center at Tyler, Tyler, TX 75708, USA; 2 Texas Lung Injury Institute, The University of Texas Health Science Center at Tyler, Tyler, TX 75708, USA

## Abstract

Influenza A virus (IAV) targets airway epithelial cells and exploits the host cell machinery to replicate, causing respiratory illness in annual epidemics and pandemics of variable severity. The high rate of antigenic drift (viral mutation) and the putative antigenic shift (reassortant strains) have raised the need to find the host cell inducible factors modulating IAV replication and its pathogenesis to develop more effective antiviral treatment. In this study, we found for the first time that transcription factor Runx3, a developmental regulator and tumor suppressor, was induced by IAV H1N1 and H3N2, viral RNA, a synthetic analog of viral double-stranded RNA (dsRNA) polyinosinic-polycytidylic acid, and type-II interferon-γ (IFNγ) in human airway epithelial cells. Whereas Runx3 was essentially not induced by type-I IFNα and type-III IFNλ, we show that Runx3 induction by IAV infection and viral RNA is mediated through the innate immune receptor MDA5 and the IκB kinase-β−NF-κB pathway. Moreover, we provide substantial evidence indicating that Runx3 plays a crucial role in airway epithelial cell apoptosis induced by IAV infection and dsRNA through the activation of extrinsic and intrinsic apoptosis pathways. Thus, we have identified Runx3 as an inducible and important transcription factor modulating IAV-induced host epithelial cell apoptosis.

Influenza is a highly contagious, acute respiratory disease that can promote exacerbations of airway and lung disorders as well as cardiovascular diseases^1^,^2^,^3^. Influenza A virus (IAV) targets airway epithelial cells and exploits the host cell machinery to replicate, causing respiratory illness in annual epidemics and every 10–50 years, pandemics of variable severity. Influenza affects all age groups, results in considerable morbidity and mortality, and exacts a formidable toll on world health and economics. Antigenic drift (viral mutation) and shift (reassortant strains) in circulating viruses cause the formation of highly virulent viruses that may escape from acquired immunity induced by the available vaccines^4^. Moreover, reports of viral resistance to current anti-influenza drugs (matrix 2 and neuraminidase inhibitors) have rapidly increased during recent years^5^,^6^. Hence, it has been proposed that identification of and targeting key inducible host cell factors modulating IAV replication and pathogenesis may provide a potential solution to these challenges^7^,^8^,^9^. One important aspect of the IAV-induced pathogenesis is host cell apoptosis, which is regarded as a cellular defense mechanism that effectively clears virus-infected cells and prevents spread of the virus^10^,^11^,^12^. However, too much or uncontrolled apoptosis could cause pulmonary architectural damage and lung dysfunction, which contributes to disease morbidity and mortality, so that the severity of IAV infection is closely related to dysregulation of lung epithelial cell apoptosis^3^,^13^,^14^.

The RUNX transcription factors play pivotal roles in normal embryonic development and neoplasia^15^,^16^. In mammals, the RUNX family consists of three members: Runx1, Runx2 and Runx3. Each RUNX member has a distinct set of functions although they recognize the same DNA binding motif. This lack of functional redundancy is due to the tightly regulated spatial and temporal expression patterns^17^. Runx1 and Runx2 are essential for hematopoiesis and osteogenesis, respectively^18^,^19^. Runx3 is closely involved in neurogenesis, thymopoiesis, gastrointestinal and lung development^19^,^20^,^21^,^22^,^23^. Runx3 knockout mice die soon after birth and display lung epithelial hyperplasia and remodeling^23^. Moreover, recent studies indicate that Runx3 can function as a tumor suppressor for a variety of cancers of gastric, breast, pancreatic, liver, lung and colon origins^24^. However, little is known about the regulation of Runx3 expression and its role in IAV infection.

To test whether Runx3 is involved in host cell responses to IAV infection, we investigated Runx3 expression and function in response to IAV infection, viral RNA and a synthetic analog of viral double-stranded RNA (dsRNA) polyinosinic-polycytidylic acid (poly(I:C)) in human airway epithelial cells. We found for the first time that Runx3 was induced by IAV H1N1 and H3N2, viral RNA, poly(I:C), and type-II interferon-γ (IFNγ) in airway epithelial cells. We also identified that Runx3 induction by IAV infection and viral RNA was mainly mediated by the innate immune receptor MDA5 and the IκB kinase (IKK)β−NF-κB pathway. Our findings further indicate that Runx3 plays an important role in airway epithelial cell apoptosis induced by IAV infection and dsRNA.

## Results

### Runx3 is induced by IAV infection in human airway epithelial cells

**Figure 1:**
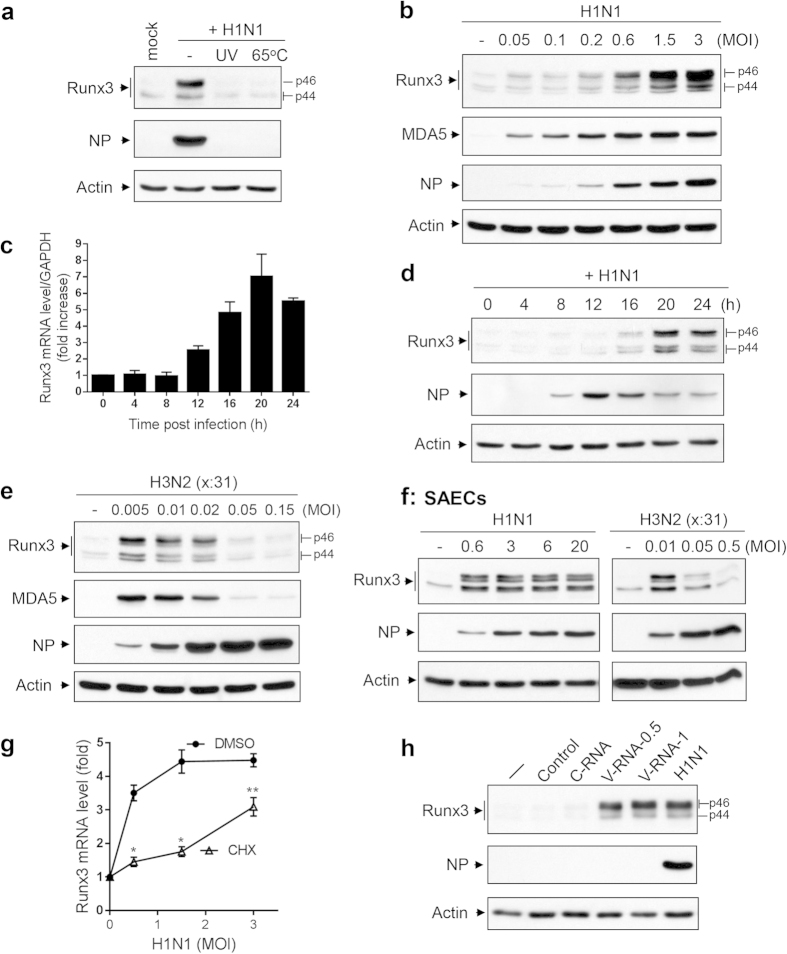
Runx3 is induced by IAV infection in airway epithelial cells. (**a**) BEAS-2B cells were treated with control PBS (mock) or infected with active H1N1 PR/8/34 strain at MOI of 1 or inactivated H1N1 viruses (UV light exposure or incubation in 65^o^ C for 30 min), grown for 24 h, and cell lysates at equal protein amounts were subjected to Western blotting with Runx3 or NP antibodies. (**b**) BEAS-2B cells were infected without (–) or with ( + ) H1N1 PR/8/34 at various MOIs of 0.05 to 3 for 24 h, and cell lysates at equal protein amounts were subjected to Western blotting with indicated antibodies. (**c,d**) BEAS-2B cells were infected with H1N1 (MOI = 1) for 0–24 h, and the kinetics of Runx3 mRNA and protein expression were determined by quantitative real-time PCR (**c**) and Western blot (**d**), respectively. (**e,f**) BEAS-2B cells (**e**) and SAECs (**f**) were infected without (–) or with ( + ) H3N2 (x:31) A/Aichi/68 or H1N1 PR/8/34 strains at different MOIs for 24 h, and cell lysates at equal protein amounts were subjected to Western blotting with indicated antibodies. (**g**) BEAS-2B cells were infected with H1N1 at various MOIs of 0.5 to 3 in the presence of control DMSO or cycloheximide (CHX, 5 μM) for 16 h. Runx3 mRNA level was measured by RT-PCR and normalized to GAPDH, and relative changes (fold) are shown. Data are means ± S.E. (n = 3). *p < 0.05; **p < 0.01 *versus* the mock-infected cells. (**h**) BEAS-2B cells were left untreated (–) or transfected with Lipofectamine 2000 plus control H_2_O (control), total RNA isolated from uninfected BEAS-2B cells (C-RNA, 0.5 μg) or from H1N1-infected BEAS-2B cells (V-RNA-0.5, 0.5 μg; V-RNA-1, 1 μg), or infected with H1N1 at MOI of 1 for 24 h. Cell lysates at equal protein amounts were subjected to Western blot analysis with indicated antibodies. Results represent the findings of three independent experiments.

Airway epithelial cells are the primary target and the principal host for respiratory viruses including IAV. We found that Runx3 protein was detected as two major p44 and p46 isoforms^25^ by a specific Runx3 antibody and that Runx3 was markedly induced by infection of IAV H1N1 PR/8/34 strain at a multiplicity of infection (MOI) of 1 in the BEAS-2B normal human bronchial epithelial cell line (Fig. 1a). Inactivated virus, generated after exposure to UV light or heat (65^o^ C) treatment, did not induce Runx3 expression; and viral nucleoprotein (NP)^26^ that is a surrogate marker of viral replication was not detected (Fig. 1a). These results indicate that Runx3 induction requires active virus replication in host cells and is not mediated solely by the initial virus-host cell interaction. We further found that Runx3 protein was induced by IAV H1N1 infection in a dose-dependent manner in BEAS-2B airway epithelial cells (Fig. 1b). Similarly, the protein levels of dsRNA receptor MDA5 and viral NP were increased in a dose-dependent manner by H1N1 infection. Interestingly, doublet bands of p44 or p46 isoforms were observed, which may represent the mobility shift of post-translationally modified Runx3 protein^27^. Quantitative real-time PCR revealed that Runx3 mRNA was induced by H1N1 12 h post-infection and reached a maximal level at 20 h in BEAS-2B cells (Fig. 1c). The time-dependent induction of Runx3 by H1N1 was confirmed at the protein level using Western blotting (Fig. 1d). We also found that Runx3 protein was induced by IAV H3N2 (x:31) A/Aichi/68 strain in BEAS-2B cells (Fig. 1e). However, unlike the H1N1 strain, H3N2 at high doses appeared to inhibit the expression of Runx3 and MDA5 in the cells. Similar to BEAS-2B cells, Runx3 can be induced by IAV H1N1 or H3N2 in primary human small airway epithelial cells (SAECs) (Fig. 1f). Additionally, Runx1 was not induced by H1N1 and Runx2 was barely detected in BEAS-2B cells (Supplementary Fig. S1a). Interestingly, we found that the level of Runx3 mRNA was significantly increased by H1N1 infection in the presence of a protein synthesis inhibitor cycloheximide (Fig. 1g), suggesting that the IAV-induced expression of Runx3 may be mediated by viral RNA. It should be noted that the magnitude of Runx3 mRNA induction by H1N1 in the presence of cycloheximide was lower than that of control cells, which may be due to the inhibition of IAV replication by cycloheximide as reported previously^28^. To further assess the role of viral RNA in Runx3 induction, we isolated total RNA from mock- or IAV-infected cells and transfected them into BEAS-2B cells. As shown in Fig. 1h, only viral RNA generated during IAV infection, but not cellular RNA, contributed to Runx3 induction. These findings indicate that transcription factor Runx3 is induced by IAV infection, likely through viral replicative RNA in airway epithelial cells.

### Runx3 is strongly induced by dsRNA poly(I:C) in airway epithelial cells

**Figure 2:**
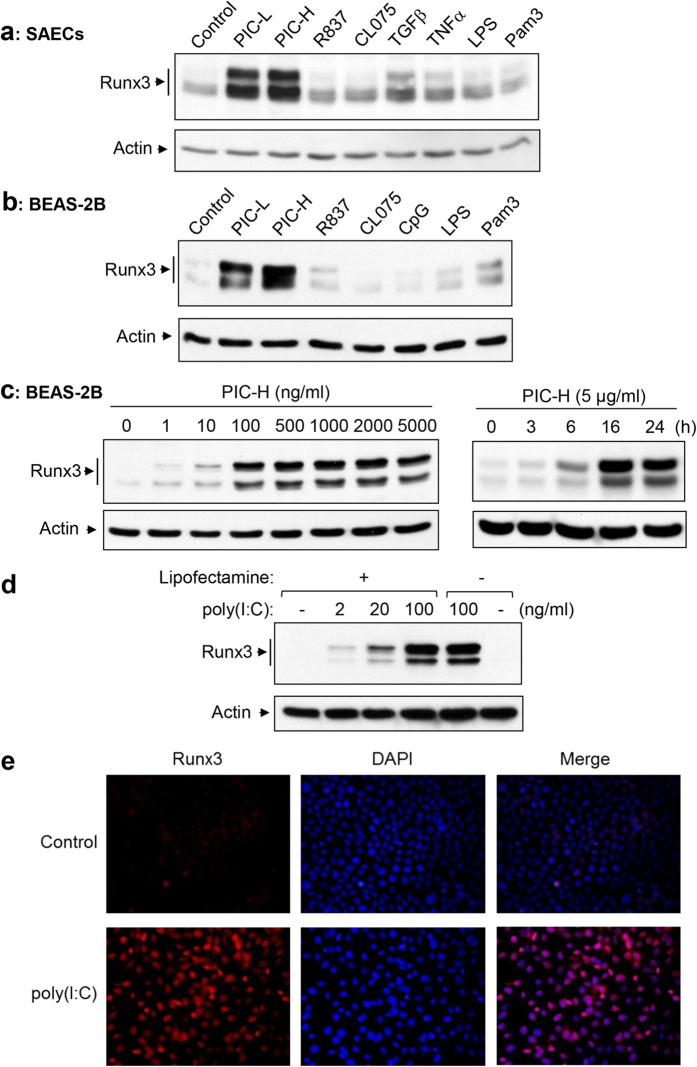
Runx3 is strongly induced by dsRNA poly(I:C) in airway epithelial cells. (**a,b**) SAECs (**a**) and BEAS-2B (**b**) cells were treated 24 h with control PBS (control), low molecular weight poly(I:C) (PIC-L, 10 μg/ml), high molecular weight poly(I:C) (PIC-H, 10 μg/ml), R837 (10 μg/ml), CL075 (10 μg/ml), TGFβ (10 ng/ml), TNFα (6 ng/ml), CpG (2 μg/ml), LPS (1 μg/ml), or Pam3CSK4 (Pam3, 10 μg/ml) as indicated. (**c**) BEAS-2B cells were treated 24 h with different doses of a high molecular weight poly(I:C) (PIC-H) (*left panels*) or with the poly(I:C) (5 μg/ml) for 0-24 h (*right panels*). (**d**) BEAS-2B cells were treated 24 h with different doses of a high molecular weight poly(I:C) complexed with (+) or without (–) lipofectamine-2000. Cell lysates at equal protein amounts from (**a–d**) were subjected to Western blot analysis with Runx3 or actin antibodies. Results shown are representative Western blots of three independent experiments. (**e**) BEAS-2B cells were treated with control PBS or poly(I:C) (2 μg/ml) for 20 h, fixed and immunostained with Runx3 monoclonal antibody or stained with DAPI as indicated. Nuclear localization of Runx3 was visualized and photographed by fluorescence microscopy. Results represent the findings of two independent experiments.

It is believed that the viral replicative intermediate dsRNA is one of the important components of infecting IAV, and the synthetic dsRNA analog poly(I:C) and viral dsRNA isolated from IAV-infected lungs are each able to induce both the local and systemic cytotoxic effects typical of influenza^29^,^30^,^31^. We thus assessed the effect of dsRNA on Runx3 expression. As shown in Fig. 2a, the level of Runx3 protein was strongly induced by low (0.2–1 kb) or high (1.5–8 kb) molecular weight dsRNA poly(I:C) in primary SAECs. Runx3 was also slightly upregulated by transforming growth factor-β (TGFβ), tumor necrosis factor-α (TNFα) and Pam3CSK4 (TLR1/2 ligand) in the cells. In contrast, other agonists including R837 (TLR7 agonist), CL075 (TLR8/7 agonist), and lipopolysaccharide (LPS, TLR4 agonist) only had minor effects. It has been shown that primary human airway epithelial cells express all TLRs except TLR8^32^. Similarly, Runx3 protein levels were markedly induced by poly(I:C) in BEAS-2B airway epithelial cells that express all TLRs^32^ (Fig. 2b). Runx3 was also induced by poly(I:C) in a dose-dependent manner in 16HBE14o- immortalized normal human bronchial epithelial cells^33^ (Supplementary Fig. S2). In contrast, neither Runx1 nor Runx2 was induced by poly(I:C) in BEAS-2B cells (Supplementary Fig. S1b). We further found that the effect of poly(I:C) on Runx3 induction could be observed at a dose as low as 1 ng/ml and reached a plateau of over 500 ng/ml in BEAS-2B cells (Fig. 2c, *left panels*) and that Runx3 induction was detected as early as 6 h after poly(I:C) treatment (Fig. 2c, *right panels*). Moreover, poly(I:C) had a similar ability to induce Runx3 expression when added directly to cell culture medium or delivered into the cells using a transfection reagent lipofectamine 2000 (Fig. 2d). This result is consistent with a previous report that poly(I:C) is internalized and functions via intracellular dsRNA receptors^34^. Additionally, an immunofluorescence analysis revealed that the poly(I:C)-induced Runx3 protein was entirely localized to cell nuclei (Fig. 2e), indicating that Runx3 functions to regulate gene expression in the nucleus. Taken together, our findings demonstrate that Runx3 can be strongly induced by dsRNA poly(I:C) in airway epithelial cells.

**Figure 3:**
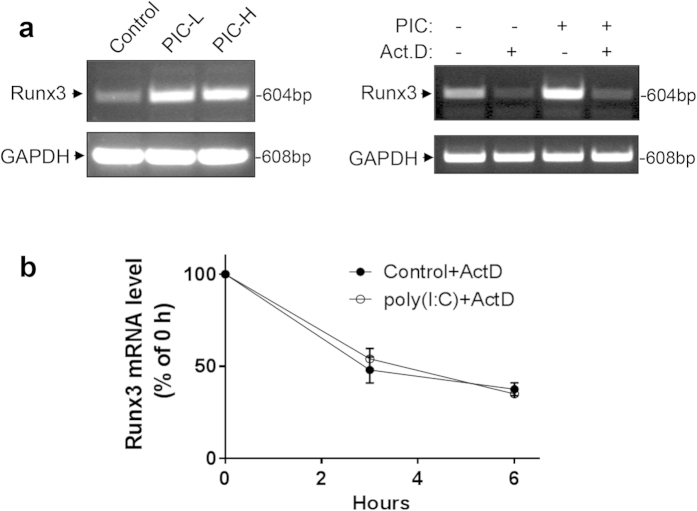
Runx3 mRNA is induced by dsRNA poly(I:C) via transcriptional regulation but not mRNA stability. (**a**) BEAS-2B cells were treated with control PBS, low molecular weight poly(I:C) (PIC-L, 10 μg/ml), or high molecular weight poly(I:C) (PIC-H, 10 μg/ml) for 16 h (*left panels*); or pretreated 2 h with (+) or without (–) actinomycin D (Act.D, 5 μM) followed by incubation with high molecular weight poly(I:C) (PIC, 10 μg/ml) for 16 h (*right panels*); and Runx3 mRNA levels are shown in a representative RT-PCR of three independent experiments. (**b**) BEAS-2B cells were treated with control PBS or high molecular weight poly(I:C) (5 μg/ml) for 16 h, then actinomycin D (Act.D, 5 μM) was added for 3 or 6 h. Relative change in Runx3 mRNA level was measured and normalized to GAPDH and is represented as percentage of Runx3 mRNA at 0 h (n = 3).

We next sought to determine whether Runx3 induction by poly(I:C) was mediated via transcriptional regulation. We found that the level of Runx3 mRNA was markedly induced by low and high molecular weight poly(I:C)s in BEAS-2B cells (Fig. 3a, *left panels*), which was abolished by pretreatment with a transcription inhibitor, actinomycin D (Fig. 3a, *right panels*). We further found that the mRNA degradation rates of Runx3 were similar in the control and poly(I:C)-treated cells (Fig. 3b). These data indicate that Runx3 is induced by poly(I:C) through the regulation of transcription but not at the level of mRNA stability.

### Runx3 induction by dsRNA poly(I:C) is primarily mediated by the MDA5/TLR3−NF-κB pathway

**Figure 4:**
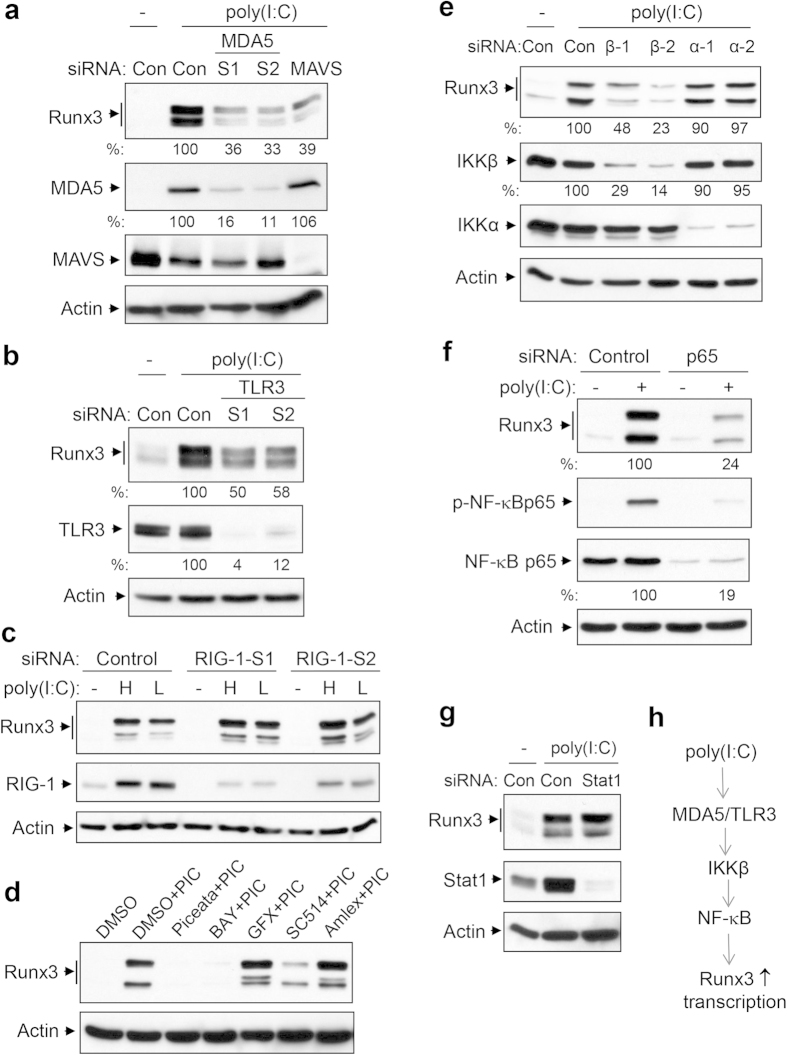
Runx3 induction by poly(I:C) is primarily mediated by MDA5/TLR3−NF-κB pathway. (**a–c**) BEAS-2B cells were transfected with 20 nM non-targeting control siRNA (Con or control), human MDA5 siRNA-1 (S1), MDA5 siRNA-2 (S2), MAVS siRNA, TLR3 siRNA-1 (S1), TLR3 siRNA-2 (S2), RIG-1 siRNA-1 (RIG-1-S1), or RIG-1 siRNA-2 (RIG-1-S2), grown for 72 h then treated with control PBS (-), high molecular weight poly(I:C) (2 μg/ml) (**a-b**), or high (H) or low (L) molecular weight poly(I:C) (2 μg/ml) (**c**) for 20 h. (**d**) BEAS-2B cells were incubated with or without poly(I:C) (PIC, 2 μg/ml) in the presence of DMSO, piceatannol (Piceata, 15 μM), BAY11-7082 (BAY, 10 μM), GFX (500 nM), SC514 (8 μM), or Amlexanol (Amlex, 10 μM) for 21 h. (**e–g**) BEAS-2B cells were transfected with 20 nM non-targeting control siRNA (Con or Control), human IKKβ siRNA-1 (β-1), IKKβ siRNA-2 (β-2), IKKα siRNA-1 (α-1), IKKα siRNA-2 (α-2), p65 or Stat1 siRNAs, grown for 60 h (**e**) or 72 h (**f,g**), then treated with control PBS (-) or high molecular weight poly(I:C) (2 μg/ml) for 20 h. Equal amounts of cell lysates from (**a-g**) were subjected to Western blot analysis with indicated antibodies. Results represent Western blots of three independent experiments. Relative changes in protein levels of Runx3, MDA5, TLR3, IKKβ or p65 were measured by densitometric analysis using ImageJ 1.47 software, normalized to actin and are represented as percentage of control siRNA (n = 3). (**h**) Signal pathway involved in Runx3 induction by poly(I:C).

The dsRNA poly(I:C) can be recognized by membrane-bound TLR3 and cytosolic RIG1-like receptors including RIG-1, MDA5, and LGP2^35^,^36^,^37^. RIG-1 preferentially binds to short (<300 bp) dsRNAs that have blunt ends and a 5′-triphosphate moiety, whereas MDA5 preferentially binds to long dsRNAs (>1000 bp) with no end specificity, such as poly(I:C) and long-scale viral replicative intermediate dsRNA^37^. We utilized an RNA interference approach to identify the pattern recognition receptors and the downstream signaling pathways responsible for Runx3 induction by poly(I:C) in BEAS-2B cells. We found that the protein level of Runx3 induced by high molecular weight poly(I:C) (1.5–8 kb) was inhibited by around 60% by MDA5 knockdown with two small interference RNAs (siRNAs) directed against different regions in human MDA5 mRNA or by knockdown of mitochondrial antiviral signaling protein (MAVS)^36^,^37^, a critical adaptor of RIG1-like receptors, compared with control siRNA (Fig. 4a). Moreover, Runx3 induction by the poly(I:C) was suppressed 42–50% by TLR3 knockdown with two functionally verified siRNAs directed against human TLR3 (Fig. 4b). In contrast, Runx3 induction by low (0.2–1 kb) or high (1.5–8 kb) molecular weight poly(I:C) was not suppressed by RIG-1 knockdown with two specific RIG-1 siRNAs (Fig. 4c). These findings indicate that MDA5 and TLR3 are two crucial innate immune receptors mediating the poly(I:C)-induced Runx3 expression in airway epithelial cells.

We next assessed the signal pathways for Runx3 induction by poly(I:C) in BEAS-2B cells. We found that Runx3 induction by poly(I:C) was abolished by two different NF-κB inhibitors piceatannol^38^ and BAY11-7082^39^ (Fig. 4d). Interestingly, Runx3 expression was attenuated by an IKKβ inhibitor SC514^40^, but not by Amlexanox^41^, an inhibitor of IKKε and TANK-binding kinase 1, or GFX109203X, a general protein kinase C inhibitor. We further found that the poly(I:C)-induced expression of Runx3 was markedly inhibited by knockdown of IKKβ, but not IKKα with two different siRNAs against human IKKβ or IKKα, respectively (Fig. 4e). Moreover, Runx3 induction by poly(I:C) was greatly suppressed by knockdown of NF-κB p65 (Fig. 4f), but not by silencing of Stat1^42^, an essential transcription factor activated by IFN (Fig. 4g). It has been well documented that TLR3 and RIG1-like receptors trigger the activation of downstream IKKα/β-NF-κB and TBK1/IKKε-IFN regulatory factor (IRF) pathways^36^,^43^,^44^,^45^, leading to the production of IFN and pro-inflammatory cytokines. We also found that knockdown of TLR3 and MDA5 inhibited the poly(I:C)-induced phosphorylation (activation) of IKKβ on Ser-177 or NF-κB p65 on Ser-536 in BEAS-2B cells (Supplementary Fig. S3). Taken together, these results indicate that MDA5 and TLR3 as well as the IKKβ−NF-κB pathway are critically involved in Runx3 induction by poly(I:C) in airway epithelial cells (Fig. 4h), although other potential pathways may also be operative.

### The IAV-induced expression of Runx3 is primarily mediated by the MDA5−NF-κB pathway

**Figure 5:**
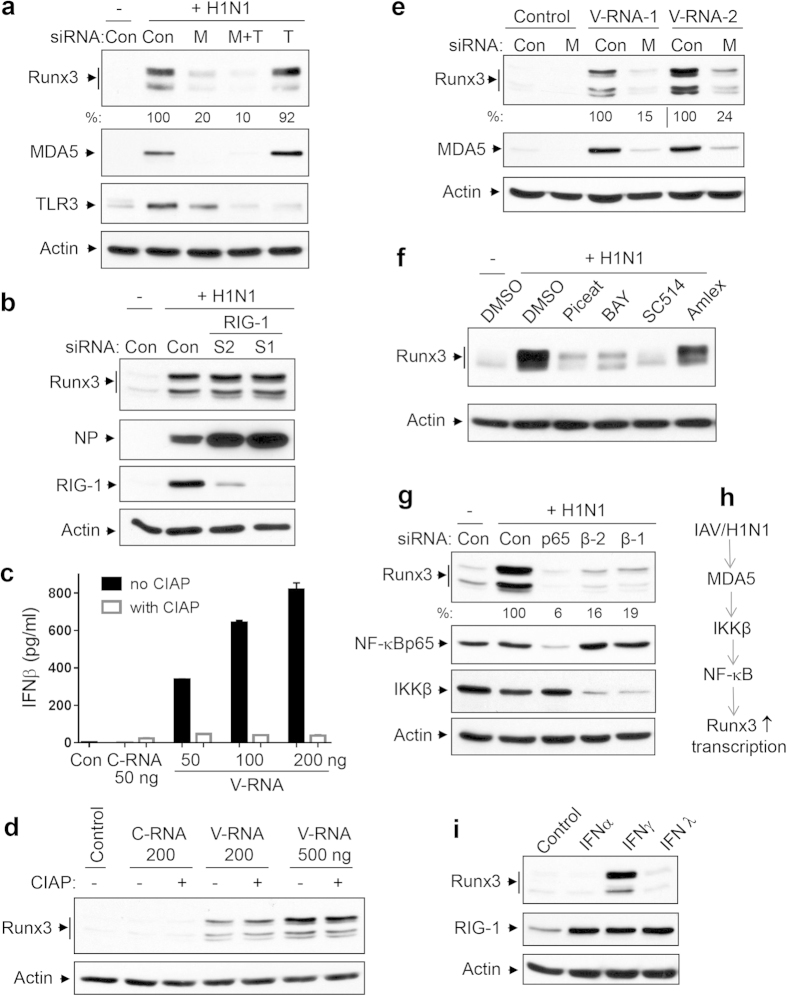
IAV-induced expression of Runx3 is primarily mediated by MDA5−NF-κB pathway. (**a**,**b**) BEAS-2B cells were transfected with 20 nM non-targeting control siRNA (Con), human MDA5 siRNA-2 (M), TLR3 siRNA-2 (T), both MDA5 and TLR3 siRNAs (M + T), RIG-1 siRNA-1 (S1), or RIG-1 siRNA-2 (S2), grown for 72 h then infected without (−) or with (+) H1N1 PR/8/34 at MOI of 1 for 24 h. (**c,d**) BEAS-2B cells were transfected with control H_2_O (Con), or different amounts of RNA isolated from uninfected BEAS-2B cells (C-RNA) or H1N1-infected BEAS-2B cells (V-RNA) treated with or without calf intestine alkaline phosphatase (CIAP). After 24 h, IFNβ released into the medium was measured by ELISA (**c**) or equal amounts of cell lysates were subjected to Western blot analysis (**d**). (**e**)BEAS-2B cells were transfected with 20 nM non-targeting control siRNA (Con) or human MDA5 siRNA-2 (M), grown for 72 h, then transfected with control H_2_O (Control) or total RNA isolated from H1N1-infected BEAS-2B cells (V-RNA-1, 300 ng; V-RNA-2, 900 ng)) for 24 h. (**f**) BEAS-2B cells were infected without (−) or with H1N1 at MOI of 1 and then incubated with DMSO, piceatannol (Piceat, 15 μM), BAY11-7082 (BAY, 10 μM), SC514 (8 μM), or Amlexanol (Amlex, 10 μM) for 24 h. (**g**) BEAS-2B cells were transfected with 20 nM non-targeting control siRNA (Con), human p65, IKKβ siRNA-1 (β-1) or IKKβ siRNA-2 (β-2), grown for 72 h, then infected with H1N1 at MOI of 1 for 24 h. (**h**) Primary signal pathway for Runx3 induction by IAV H1N1. (**i**) BEAS-2B cells were treated with control PBS, IFNα (300 U/ml), IFNγ (200 ng/ml), or IFNλ (200 ng/ml) for 24 h. Equal amounts of cell lysates from (**a–i**) were subjected to Western blot analysis with indicated antibodies. Results represent Western blots of three independent experiments. Relative changes in protein levels of Runx3 of three independent experiments were measured by densitometric analysis using ImageJ 1.47 software and normalized to actin and are represented as percentage of control siRNA (n = 3).

We utilized the functionally verified siRNAs of MDA5, TLR3 or RIG-1 to determine the pattern recognition receptors responsible for Runx3 induction by IAV in BEAS-2B cells. As shown in Fig. 5a, we found that Runx3 induction by IAV H1N1 was suppressed 80% by knockdown of MDA5 in BEAS-2B cells. In contrast, knockdown of TLR3 or RIG-1 had only minor effects on Runx3 expression (Fig. 5a,b). RIG-1 preferentially binds to viral RNA with a 5′-triphosphate moiety^37^ and plays an essential role in type-I IFN production and antiviral activity^46^. Indeed, knockdown of RIG-1 enhanced IAV replication as evidenced by the increased expression of viral NP protein (Fig. 5b). To verify whether RIG-1 is involved in Runx3 induction by IAV, we transfected BEAS-2B cells with the isolated total RNA from mock- or IAV-infected cells that had been treated with calf intestine alkaline phosphatase (CIAP) to remove the 5′-triphosphate moiety. As expected, treatment of IAV viral RNA with CIAP abolished IFNβ production (Fig. 5c). Interestingly, only viral RNA generated during IAV infection, but not cellular RNA, contributed to Runx3 induction and the effect was apparently not affected by CIAP treatment (Fig. 5d). Moreover, Runx3 induction by IAV viral RNA was markedly suppressed by knockdown of MDA5 in BEAS-2B cells (Fig. 5e), but was largely not affected by knockdown of TLR3 or RIG-1 (Supplementary Fig. S4). These results further indicate that MDA-5, but not TLR3 or RIG-1, is primarily involved in Runx3 induction by IAV or IAV viral RNA.

We next assessed the signal pathways responsible for Runx3 induction by IAV in BEAS-2B cells. We found that similar to Runx3 induction by dsRNA poly(I:C), IAV-induced expression of Runx3 was markedly suppressed by two different NF-κB inhibitors piceatannol^38^ and BAY11-7082^39^ and an IKKβ inhibitor SC514^40^ (Fig. 5f). Furthermore, the IAV-induced expression of Runx3 was essentially abolished by silencing of NF-κB p65 and was markedly inhibited by knockdown of the NF-κB upstream activator IKKβ with two different siRNAs (Fig. 5g). Collectively, our findings indicate that IAV induces Runx3 expression mainly through MDA5 and the IKKβ−NF-κB pathway in airway epithelial cells (Fig. 5h). Since IAV infection induces the expression and secretion of type-I and type-III IFNs in airway epithelial cells^32^, we assessed the effects of different types of IFNs on Runx3 expression in BEAS-2B cells. Interestingly, we found that Runx3 was induced by type-II IFNγ, but essentially not by type-I IFNα and type-III IFNλ, although they all induced RIG-1 expression at comparable levels (Fig. 5i). This result suggests that autocrine type-I and type-III IFNs may not contribute to Runx3 induction by IAV infection in airway epithelial cells.

### Runx3 plays an important role in airway epithelial cell apoptosis induced by IAV and dsRNA poly(I:C)

**Figure 6:**
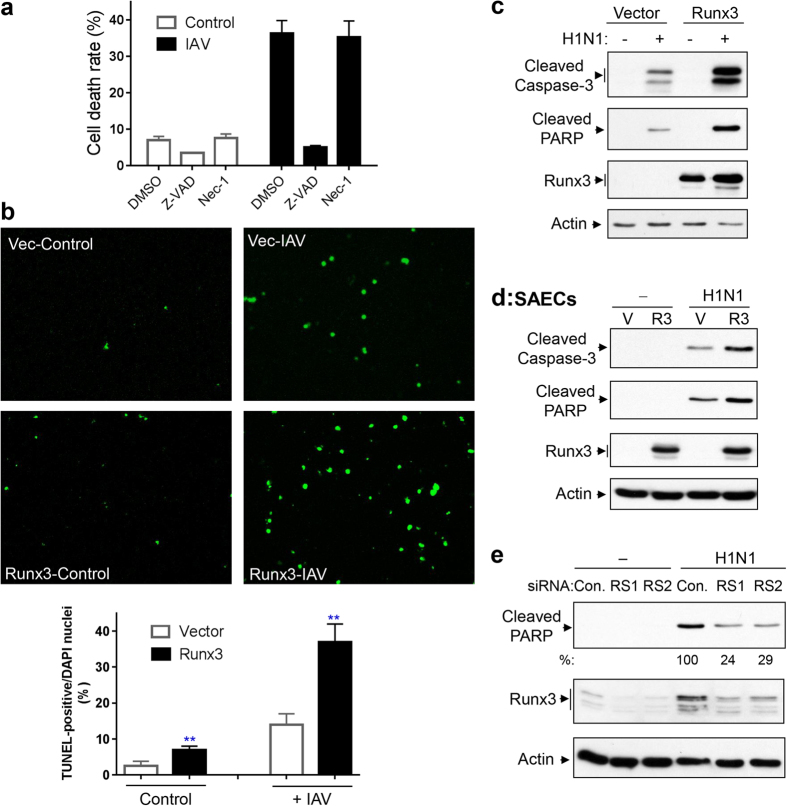
Runx3 is critical for airway epithelial cell apoptosis induced by IAV infection. (**a**) BEAS-2B cells were infected with H1N1 PR/8/34 at MOI of 3 for 24 h in the presence or absence of Z-VAD-FMK (Z-VAD, 20 μM) or necrostatin-1 (Nec-1, 50 μM). Detached and adherent cells were collected separately and counted with trypan blue by using a TC20 automated cell counter and cell death rate (detached dead cells over total detached and adherent cells) was shown. Data are means ± S.E. (n = 3). (**b**) BEAS-2B cells were infected with recombinant adenovirus containing vector alone (Vec) or Runx3, grown for 60 h, then infected with (+) IAV H1N1 at MOI of 1 or treated with control PBS (Control) for 24 h. Representative images (final magnification: ×200) of TUNEL staining and cell apoptotic rate (%) from three independent experiments are shown. Apoptotic rate was determined as TUNEL-positive cells (green color) over total DAPI-stained nuclei by using ImageJ 1.47 software. Data are means ± S.E. (n = 3). **p < 0.01 *versus* cells expressing vector alone. (**c,d**) BEAS-2B cells (**c**) and primary SAECs (**d**) were infected with recombinant adenovirus containing vector alone (Vector or V) or Runx3 (Runx3 or R3), grown for 60 h, then infected without (−) or with ( + ) H1N1 at MOI of 1 for 24 h. Cell lysates at equal protein amounts were subjected to Western blotting with indicated antibodies. (**e**) BEAS-2B cells were transfected with 20 nM control siRNA (Con.), Runx3 siRNA-1 (RS1), or Runx3 siRNA-2 (RS2), grown for 72 h, then infected with H1N1 at MOI of 1 for 24 h. Equal amounts of cell lysates were subjected to Western blotting with indicated antibodies. Results represent Western blots of three independent experiments. Relative changes in cleaved PARP in (**e**) were measured by densitometric analysis and normalized to actin and are represented as percentage of control siRNA (n = 3).

As shown in Fig. 6a, we determined the detached dead cells from culture with trypan blue using a TC20 automated cell counter and found that the cellular death rate (detached dead cells over total detached and adherent cells) was greatly enhanced by infecting BEAS-2B cells with IAV H1N1. We further found that the IAV-induced cell death was abolished by a general caspase inhibitor Z-VAD-FMK^47^, but was essentially unaffected by a necroptosis inhibitor necrostatin-1^48^. These results indicate that, in agreement with previous reports^10^,^11^,^12^, the IAV-induced airway epithelial cell death is mediated by apoptosis. DNA fragmentation represents a characteristic of late stage apoptosis that can be detected by TUNEL assay^49^. TUNEL staining (green color) revealed that infection of BEAS-2B cells with H1N1 induced cell apoptosis, and the apoptotic rate was markedly augmented (2.6-fold) by Runx3 overexpression (Fig. 6b and supplementary Fig. S5). Overexpression of Runx3 also increased the basal cellular apoptotic rate. To understand the molecular mechanisms of Runx3 in mediating apoptosis, we determined the effect of Runx3 on the activation (cleavage) of caspase-3^50^, a primary executioner of apoptosis, and the cleavage of DNA repair enzyme poly (ADP-ribose) polymerase (PARP)^51^, one of the main cleavage targets of caspase-3. We found that H1N1 infection induced the cleavage (activation) of caspase-3 and PARP and that the effects were markedly enhanced by Runx3 overexpression in BEAS-2B cells (Fig. 6c). Similarly, overexpression of Runx3 also augmented the cleavage (activation) of caspase-3 and PARP in primary SAECs (Fig. 6d). To confirm if Runx3 mediates cellular apoptosis in response to IAV, we knocked down Runx3 with two specific siRNAs that target different regions in human Runx3 mRNA and determined the effects on apoptosis by measuring PARP cleavage. As shown in Fig. 6e, the H1N1-induced cleavage of PARP was markedly suppressed by Runx3 knockdown in BEAS-2B cells. These novel findings demonstrate that Runx3 plays a crucial role in airway epithelial cell apoptosis induced by IAV infection.

**Figure 7:**
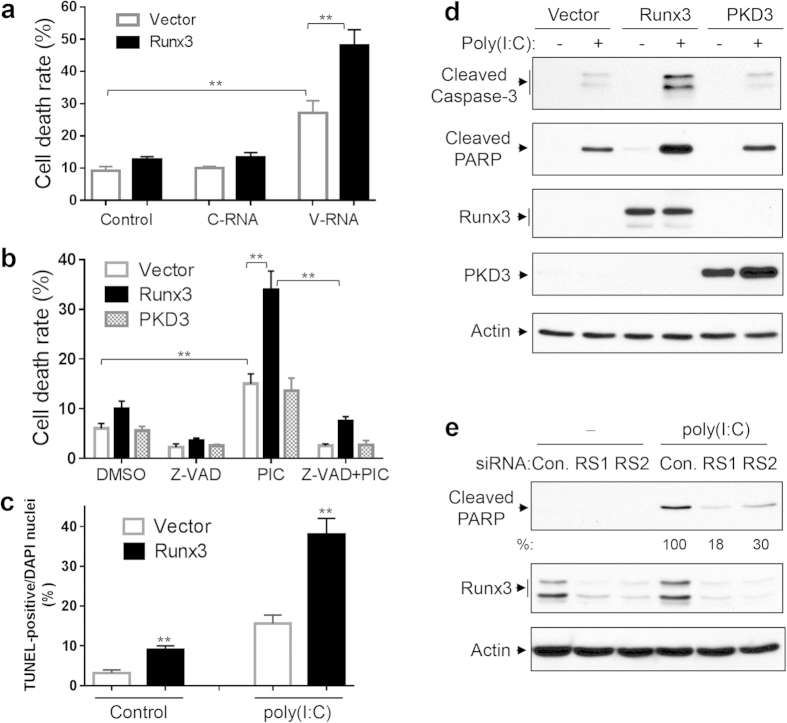
Runx3 promotes airway epithelial cell apoptosis induced by IAV viral RNA and dsRNA poly(I:C). (**a–d**) BEAS-2B cells were infected with recombinant adenovirus containing vector alone, Runx3 or PKD3, and grown for 60 h. (**a**) The cells were then transfected with control H_2_O (control), or total RNA isolated from uninfected BEAS-2B cells (C-RNA, 0.5 μg) or H1N1-infected BEAS-2B cells (V-RNA, 0.5 μg) for 24 h. The cellular death rate (detached dead cells over total detached and adherent cells) was determined by using a TC20 automated cell counter with trypan blue. (**b**) The cells were then left untreated or treated 4 h with high molecular weight poly(I:C) (2 μg/ml) in the presence or absence of Z-VAD-FMK (Z-VAD, 20 μM) and the death rate of the cells was determined. (**c**) The cells were treated with control PBS (Control) or poly(I:C) (2 μg/ml) for 4 h and cellular apoptosis was assessed by TUNEL assay. Cell apoptotic rate was determined as TUNEL-positive cells over total DAPI-stained nuclei (supplementary Fig. S5) by using ImageJ 1.47 software. All Data in (**a–c**) are means ± S.E. of three independent experiments. **p < 0.01 *versus* control or control vector. (**d**) The cells were treated with control PBS (-) or high molecular weight poly(I:C) (2 μg/ml) for 4 h and cell lysates at equal protein amounts were subjected to Western blotting with indicated antibodies. (**e**) BEAS-2B cells were transfected with 20 nM control siRNA (con.), Runx3 siRNA-1 (RS1), or Runx3 siRNA-2 (RS2), grown for 72 h, then treated with control PBS (-) or poly(I:C) (2 μg/ml) for 4 h. Equal amounts of cell lysates were subjected to Western blotting with indicated antibodies. Relative changes in cleaved PARP of three independent experiments were measured by densitometric analysis and normalized to actin and are represented as percentage of control siRNA (n = 3). Results shown are representative Western blots of three independent experiments.

We then asked whether Runx3 promoted IAV-induced apoptosis by enhancing the apoptosis-inducing effects of viral RNA or dsRNA. We first determined the effect of Runx3 overexpression on cell death induced by total RNA isolated from mock- or IAV-infected cells. As shown in Fig. 7a, viral RNA generated during IAV infection, but not the control cellular RNA, significantly induced cell death and the effect was indeed augmented by Runx3 overexpression. We further found that dsRNA poly(I:C) significantly increased cell death and the cellular death rate was also enhanced by Runx3 overexpression, which was abolished by a general caspase inhibitor Z-VAD-FMK^47^ (Fig. 7b). In contrast, overexpression of protein kinase D3 (PKD3), which was used as an experimental control, had a minor effect on the cellular death rate (Fig. 7b) and apoptosis by measuring the cleavage of caspase-3 and PARP (Fig. 7d). Overexpression of Runx3 and PKD3 was verified by Western blots in Fig. 7d (*3*^*rd*^ *& 4*^*th*^ *panels*). Moreover, TUNEL staining revealed that Runx3 overexpression apparently augmented basal and the poly(I:C)-induced cell apoptotic rate (Fig. 7c). The images of TUNEL staining are shown in supplementary Fig. S6. Consistent with IAV-induced apoptosis, we found that the poly(I:C)-induced cleavage (activation) of caspase-3 and PARP was augmented by Runx3 overexpression (Fig. 7d), but was markedly suppressed by Runx3 knockdown with two different siRNAs in BEAS-2B cells (Fig. 7e). These findings demonstrate that Runx3 positively mediates airway epithelial cell apoptosis induced by IAV viral RNA and dsRNA poly(I:C).

**Figure 8:**
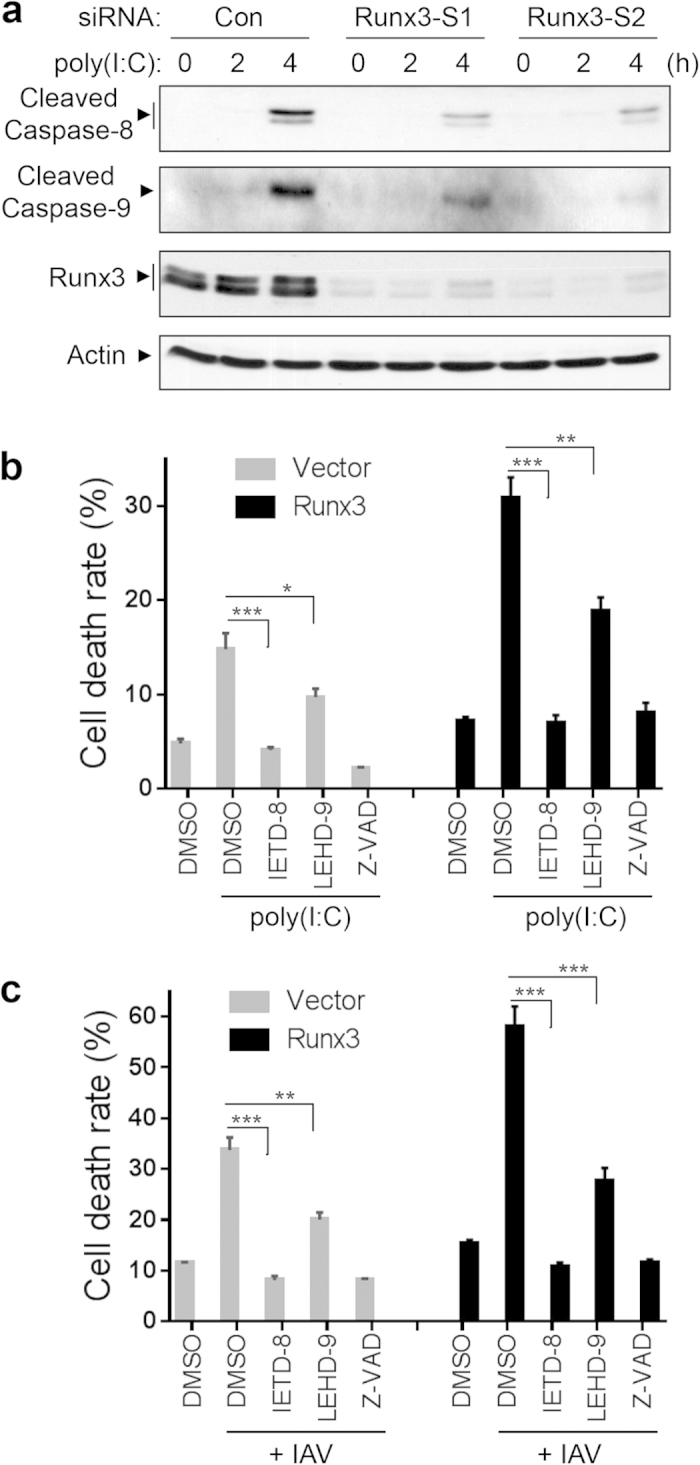
Both the extrinsic and intrinsic pathways are involved in Runx3-promoted cell apoptosis by dsRNA poly(I:C) and IAV infection. (**a**) Runx3 is required for the activation of caspase-8 and caspsas-9 by poly(I:C). BEAS-2B cells were transfected with 20 nM control siRNA (Con.), Runx3 siRNA-1 (Runx3-S1), or Runx3 siRNA-2 (Runx3-S2), grown for 72 h, then treated with high molecular weight poly(I:C) (2 μg/ml) for 0, 2 or 4 h. Equal amounts of cell lysates were subjected to Western blotting with specific antibodies against cleaved caspase-8, cleaved caspase-9, Runx3 or action as indicated. (**b,c**) BEAS-2B cells were infected with recombinant adenovirus containing vector alone or Runx3 and grown for 60 h. The cells were then left untreated or treated with poly(I:C) (2 μg/ml) for 4 h (**b**) or infected with (+) IAV H1N1 at MOI of 3 for 24 h (**c**) in the presence or absence of caspase-8 inhibitor Z-IETD-FMK (IETD-8, 20 μM), caspace-9 inhibitor Z-LEHD-FMK (LEHD-9, 20 μM), or a general caspase inhibitor Z-VAD-FMK (Z-VAD, 20 μM), and cell death rate was assessed as in Figure 7. All data are means ± S.E. of triplicates. *p < 0.05; **p < 0.01; ***p < 0.001 *versus* caspase-8 or caspase-9 inhibitors. Results represent the findings of three independent experiments.

It has been shown that both extrinsic (death receptor/caspase-8) and intrinsic (mitochondrial/caspase-9) apoptosis pathways are activated by dsRNA poly(I:C) and are involved in the poly(I:C)-induced apoptosis of endothelial and lung cancer cells^52^,^53^. In agreement with previous reports, we found that treatment of BEAS-2B cells with poly(I:C) triggered the activation of caspase-8 and caspase-9, resulting in the appearance of cleaved fragments p41/43 or p35/37, respectively (Fig. 8a). We further found that the poly(I:C)-induced activation of caspase-8 and caspase-9 were markedly suppressed by knockdown of Runx3 with two different siRNAs, indicating that Runx3 is required for the activation of caspase-8 and caspase-9 apoptosis pathways. We then utilized specific peptide inhibitors for caspase-8 or caspase-9 and assessed the involvement of these apoptosis pathways in poly(I:C)- or H1N1-induced apoptosis in BEAS-2B cells overexpressing Runx3 or control vector. As shown in Fig. 8b,c, the poly(I:C)- and H1N1-induced cellular death was abolished by a general caspase inhibitor Z-VAD-FMK^47^ and the specific caspase-8 inhibitor Z-IETD-FMK, but was partially inhibited by the specific caspace-9 inhibitor Z-LEHD-FMK. Similarly, the enhanced cell death rates induced by poly(I:C) and H1N1 in Runx3-overexpressing cells were also abolished by the general caspase inhibitor Z-VAD-FMK and the caspase-8 inhibitor, and partially attenuated by the caspase-9 inhibitor. Collectively, these results indicate that Runx3 is required for the activation of both extrinsic caspase-8 and intrinsic caspsas-9 apoptosis pathways that may promote airway epithelial cell apoptosis induced by dsRNA poly(I:C) and IAV infection.

## Discussion

IAV infects all age groups and poses a global health and economic threat. The emergence of viral resistance to current anti-influenza drugs and putative pandemic strains has raised the need to find the host cell inducible factors modulating IAV replication and pathogenesis for antiviral treatment^7^,^8^,^9^. In the present study, we report a novel finding that induction of transcription factor Runx3 is a host airway epithelial cell response to IAV infection, viral RNA, dsRNA poly(I:C), and type-II IFNγ and that Runx3 plays a crucial role in the epithelial cell apoptosis induced by IAV and dsRNA. We further show that Runx3 induction by IAV infection and viral RNA is mediated through the innate immune receptor MDA5 and IKKβ−NF-κB pathway. Our findings identify Runx3 as an inducible and important transcription factor that can modulate the IAV-induced host airway epithelial cell apoptosis.

Runx3 is involved in neurogenesis, thymopoiesis, gastrointestinal and lung development^19^,^20^,^21^,^22^,^23^. Recent studies also indicate that Runx3 can function as a tumor suppressor for a variety of cancers of gastric, breast, pancreatic, liver, lung and colon origins^24^. However, little is known about the regulation of Runx3 expression. The first finding from this study is the induction of Runx3 by IAV infection, viral RNA, dsRNA poly(I:C) and IFNγ in airway epithelial cells. This is the first report that Runx3 can be upregulated in epithelial cells as far as we know. The induction of Runx3 by IAV required active virus replication in host cells; and Runx3 was induced in a dose-dependent manner by H1N1 PR/8/34 and H3N2 (x:31) A/Aichi/68 strains. However, unlike the H1N1 strain, the H3N2 (x:31) A/Aichi/68 strain at high doses inhibited Runx3 expression. This may be because the nonstructural protein 1 of H1N1 PR8/34 strain lacks a functional binding domain to polyadenylation stimulating factor 30 to inhibit host gene expression^54^. We also found that Runx3 mRNA level was significantly increased by H1N1 infection in the presence of a protein synthesis inhibitor cycloheximide, and that viral RNA generated during IAV infection, but not cellular RNA, contributed to Runx3 induction. Furthermore, we have demonstrated that Runx3 is strongly induced by dsRNA poly(I:C) through the regulation of transcription but not at the level of mRNA stability. Hence, our findings indicate that Runx3 is induced by IAV infection likely through viral replicative RNA or dsRNA in airway epithelial cells. It has been shown IAV infection results in accumulation of viral replicative intermediate dsRNAs, which can be recognized by innate immune receptors, such as membrane-bound TLR3 and cytosolic RIG1-like receptors including RIG-1, MDA5, and LGP2^35^,^36^,^37^. TLR3 localizes to both the cell membrane surface and intracellular endosomal compartments of epithelial cells^32^,^55^ and mediates inflammatory cytokine production by IAV^56^. RIG-1 preferentially binds to short (<300 bp) dsRNAs that have blunt ends and a 5′-triphosphate moiety^37^ and plays an essential role in type-I IFN production and antiviral activity^46^. On the other hand, MDA5 preferentially binds to long dsRNA (>1000 bp) with no end specificity and thus can bind poly(I:C) and long-scale viral replicative intermediate dsRNA^37^. Interestingly, we found that Runx3 induction by IAV infection and viral RNA was primarily mediated by MDA5. Whereas Runx3 induction by direct addition of the synthetic dsRNA analog poly(I:C) was mediated by MDA5 and TLR3. A previous study shows that poly(I:C) is internalized and functions via intracellular dsRNA receptors^34^. Consistent with this report, we found that poly(I:C) had a similar ability to induce Runx3 expression when added directly to cell culture medium or delivered into the cells. These results suggest that the internalized poly(I:C) may activate cytosolic MDA5 and endosomal TLR3 to induce Runx3 expression, while long-scale dsRNA derived from virus replication may only activate MDA5 for Runx3 induction. They also suggest that the entry of long scale viral dsRNA into the endosome through autophagic processes may be limited. Moreover, we have provided substantial evidence indicating that IKKβ−NF-κB pathway is critically involved in Runx3 induction by IAV infection and dsRNA poly(I:C) in airway epithelial cells. Additionally, we found that Runx3 was induced by type-II IFNγ but essentially not by type-I IFNα and type-III IFNλ, and that silencing of Stat1^42^ that is an essential transcription factor downstream of IFN did not affect Runx3 expression by poly(I:C). These findings indicate that Runx3 induction by IAV infection or dsRNA in airway epithelial cells is independent of autocrine type-I and type-III IFNs. Sequence analysis reveals that Runx3 promoter area (+1 to −1424) contains three sites of NF-κB core binding sequence (GGGRNWYYCC) and one gamma activation sequence (TTC(N_2–4_)GAA).

Another important finding from this study is that Runx3 plays a crucial role in airway epithelial cell apoptosis induced by IAV infection and dsRNA. It has been shown that IAV infection induces cell apoptosis *in vitro* and in animal tissues and that the apoptotic cells along with the replicative virus within the cell are phagocytosed, digested and killed by macrophages and neutrophils, hence cellular apoptosis is regarded as a host defense mechanism to effectively clear the virus-infected cells to prevent spread of the virus^10^,^11^,^12^,^57^. Under this scenario, Runx3 may function as a host antiviral factor as Runx3 promotes airway epithelial cell apoptosis in response to IAV infection and dsRNA. However apoptosis is a double-edged sword for IAV infection. Too much or uncontrolled apoptosis could cause pulmonary tissue damage and lung dysfunction, which contributes to the disease morbidity and mortality, so upregulation of Runx3 may enhance the IAV-induced pathogenesis and the disease severity by promoting host cell apoptosis and tissue injury. The role of Runx3 in lung epithelial cell response and disease progression to IAV infection *in vivo* merits further investigation.

In summary, we have identified Runx3 as an inducible and crucial transcription factor that can modulate airway epithelial cell apoptosis in response to IAV infection, viral RNA and dsRNA poly(I:C). Our study also opens new fields of investigation of the upregulation of tumor suppressor Runx3 in carcinoma cells or in other forms of acute lung injury and repair, in which lung epithelial cell apoptosis is generally a prominent feature.

## Methods

### Antibodies and reagents

Antibodies specifically against Runx3 (Cat. No. 9647 and 13089), Runx2, TLR3, MDA5, IKKα, IKKβ, PKD3, cleaved caspase-3, −8, −9, cleaved PARP (Asp214), Stat1, phospho-IKKα (Ser176)/IKKβ (Ser177), phospho-p65 (Ser-536) were from Cell Signaling Technology. Runx1 and NFκB p65 antibodies were from Santa Cruz Biotechnology. RIG-1 antibody was from OriGene Technology. Actin antibody, actinomycin D and LPS (*Escherichia coli* 0111:B4) were from Sigma. TLR ligands Type B CpG oligonucleotide (ODN 2006), low and high molecular weight poly(I:C), R837, CL075, and Pam3CSK4 were from *Invivo*Gen. Recombinant TGFβ, TNFα, and specific peptide inhibitors for caspase-3 (Z-VAD-FMK), caspase-8 (Z-IETD-FMK) and caspase-9 (Z-LEHD-FMK) were from R & D Systems. IFNβ ELISA kit and IFNα were from PBL Assay Science, and IFNγ and IFNλ were from eBioscience. TUNEL Apo-Green detection kit was from Biotool. Bay 11-7082, Piceatannol and GFX109203X were from EMD Millipore. Amlexanox, SC514 and necrostatin-1 were from Tocris Bioscience. Clarity Western ECL substrate was from Bio-Rad.

### Cell culture and treatment

SAECs were obtained from American Type Culture Collection (ATCC) and Lonza, cultured in airway epithelial cell growth medium (ATCC) and used for experiments within 3 passages. BEAS-2B cells were obtained from ATCC and cultured in airway epithelial cell growth medium. 16HBE14o- human bronchial epithelial cells^33^ were kindly provided by Dr. Dieter Gruenert (University of California at San Francisco) and cultured in Dulbecco’s Modified Eagle Medium (DMEM) supplemented with 10% fetal bovine serum. For agonist treatment, epithelial cells were washed twice with Hanks’ balanced salt solution and then incubated in bronchial or small airway epithelial basal medium (BEBM or SABM, Lonza) or DMEM serum-free medium with indicated agonists.

### Virus infection

BEAS-2B or SAECs grown until subconfluence or pretreated were washed twice with RPMI-1640, infected with IAV H1N1 PR/8/34 or H3N2 (x:31) A/Aichi/68 strains (Charles River) in BEBM at an appropriate MOI for 1 h at 37^o^ C, then incubated in airway epithelial cell growth medium without aspirating the viruses for 1 h to facilitate IAV entry into cells. The cells were washed once with RPMI-1640 and incubated in BEBM or SABM for 24 h. For adenoviral vector-mediated expression of Runx3, BEAS-2B cells were infected with recombinant adenovirus expressing control vector, human Runx3 or PKD3 (SignaGen Laboratories) at a MOI of 5.

### Western blot analysis

Western blot analysis was performed essentially as we described previously^58^. The membrane was probed with various primary antibodies as indicated and detected with horseradish peroxidase-conjugated secondary antibodies and Clarity Western ECL substrate (Bio-Rad).

### RNA isolation, RT-PCR and quantitative real-time PCR

Total RNA was isolated using the RNeasy RNA isolation kit according to the manufacturer’s protocol (Qiagen). RT-PCR primers were designed with Oligo6 software (Molecular Biology Insights) as follows: Runx3 (expected product of 604 bp), 5′-TGT GAT GGC AGG CAA TGA CGA-3′ (forward) and 5′-CGG GAG GTA GGT ATG GTG-3′ (reverse); and internal control glyceraldehyde-3-phosphate dehydrogenase (GAPDH) (608 bp), 5′-CGC TGA GTA CGT CGT GGA G-3′(forward) and 5′-GAG GAG TGG GTG TCG CTG TTG-3′ (reverse). RT-PCR conditions were 50 ^o^C for 30 min and 95^o^ C for 15 min, followed by 30 cycles of 94 ^o^ C for 30 sec, 55 ^o^C for 1 min, and 72 ^o^C for 1 min, followed by 72 ^o^C for 10 min. The relative change in mRNA was measured by densitometric analysis and normalized to GAPDH. For quantitative real-time PCR, RNA (300 ng) was reverse transcribed in a final volume of 20 μl using QuantiTect Reverse Transcription kit (Qiagen) according to the manufacturer’s protocol. Real-time PCR was done using StepOnePlus Real-Time PCR Systems (Applied Biosystems) and PCR reactions were conducted in a final volume of 20 μl containing: 1 × QuantiTect SYBR Green PCR Master Mix (Qiagen), primers of human Runx3 (Cat. No. QT00040306, Qiagen) or GAPDH (Cat. No. QT00079247, Qiagen) and cDNA as template. Amplification conditions were: 95 °C (15 min), 40 cycles of 94 °C (15 s), 55 °C (30 s), 72 °C (30 s). All reactions were run in triplicate, averaged, and normalized to GAPDH to quantify the relative gene expression from that of the mock-infected control using the 2^−ΔΔCT^ method.

### SiRNA and siRNA transfection

AllStars non-targeting negative control siRNA (no. 1027280), Runx3 siRNA-1 (no. SI00709051), TLR3 siRNAs (no. SI02655156 and SI00050043), MDA5 siRNAs (no. SI03649037 and SI04133717), RIG-1 siRNAs (no. SI04181821 and SI04208673), MAVS siRNA (no. SI04268761), NF-κB p65 siRNA (no. SI00301672), IKKα siRNAs (no. SI00605115 and SI04379333), IKKβ siRNAs (no. SI00300545 and SI05460700), and Stat1 siRNA (no. SI02662324) were from Qiagen. Runx3 siRNA-2 (no. sc-37679) was from Santa Cruz Biotechnology. For siRNA transfection, BEAS-2B cells were seeded into different plates for 24 h to reach 50–70% confluence, and then siRNA was transfected into the cells in a final concentration of 20 nM by using Lipofectamine 2000 and Opti-MEM I reduced serum medium according to the manufacturer’s protocol (Life Technologies). After 24 h, the medium was changed to fresh growth medium and the cells were grown for 60 or 72 h until agonist treatment. The silencing effects of siRNAs were confirmed by Western blot analysis.

### Dephosphorylation of viral or cellular RNA

Total RNA was isolated from BEAS-2B cells infected with H1N1 for 24 h (viral RNA) or from uninfected cells (cellular RNA) by using the RNeasy RNA isolation kit according to the manufacturer’s protocol (Qiagen). The calf intestine alkaline phosphatase (CIAP) (Life Technologies) was used to dephosphorylate viral 5′ triphosphate RNA in a 40 μl volume containing 20 μg RNA, 10 U CIAP, 40 U RiboLock RNase inhibitor (Life Technologies) and reaction buffer as described previously^59^. The reaction mixture was incubated for 3 h at 42^o^ C and then the RNA was isolated using the RNeasy mini kit from Qiagen. RNA treated with dilution buffer replacing CIAP was used as an experimental control. These viral or cellular RNAs were transfected into the cells by using Lipofectamine 2000 and Opti-MEM I reduced serum medium according to the manufacturer’s protocol (Life Technologies).

### ELISA assay

IFNβ in cell culture supernatants was measured by using an ELISA kit according to the manufacturer’s protocol (PBL Assay Science).

### TUNEL apoptosis assay and fluorescence microscopy

BEAS-2B cells grown on cover slips were fixed in 4% paraformaldehyde in cold phosphate-buffered saline (PBS) for 25 min at 4 °C, washed twice with PBS and permeabilized with 0.1% triton X-100 in PBS for 5 min at room temperature. The cells were then incubated with TUNEL assay reaction mixture containing FITC-12-dUTP according to the manufacturer’s protocol (Biotool). Fluorescence was visualized and images were captured by fluorescence microscopy. Adobe Photoshop CS6 software was used for image processing.

### Statistical analysis

Data are expressed as means ± SE. A Student’s *t* test was used for statistical analysis. p < 0.05 is considered statistically significant.

## Additional Information

**How to cite this article**: Gan, H. *et al.* Transcription Factor Runx3 Is Induced by Influenza A Virus and Double-Strand RNA and Mediates Airway Epithelial Cell Apoptosis. *Sci. Rep.* **5**, 17916; doi: 10.1038/srep17916 (2015).

## Supplementary Material

Supplementary Information
