# Supplementary material for: Transcription Factor Runx3 Is Induced by Influenza A Virus and Double-Strand RNA and Mediates Airway Epithelial Cell Apoptosis
Source: Sci Rep. 2015 Dec 8;5:17916. doi: 10.1038/srep17916 (PMC4672321; doi:10.1038/srep17916)
Supplement: Supplementary Information [file srep17916-s1.pdf]

Transcription Factor Runx3 Is Induced by Influenza A Virus and Double-Strand RNA and Mediates Airway Epithelial Cell Apoptosis

Huachen Gan[1], Qin Hao[1], Steven Idell[1,2] & Hua Tang[1*]

[1]Department of Cellular and Molecular Biology, The University of Texas Health Science Center at Tyler, Tyler, TX 75708, USA, [2]Texas Lung Injury Institute, The University of Texas Health Science Center at Tyler, Tyler, TX 75708, USA.

∗ Address correspondence to: Hua Tang, Department of Cellular and Molecular Biology, The University of Texas Health Science Center at Tyler, 11937 US Highway 271, Tyler, Texas 75708 (E-mail: hua.tang@uthct.edu).

Running title: Runx3 induction and function in IAV infection.

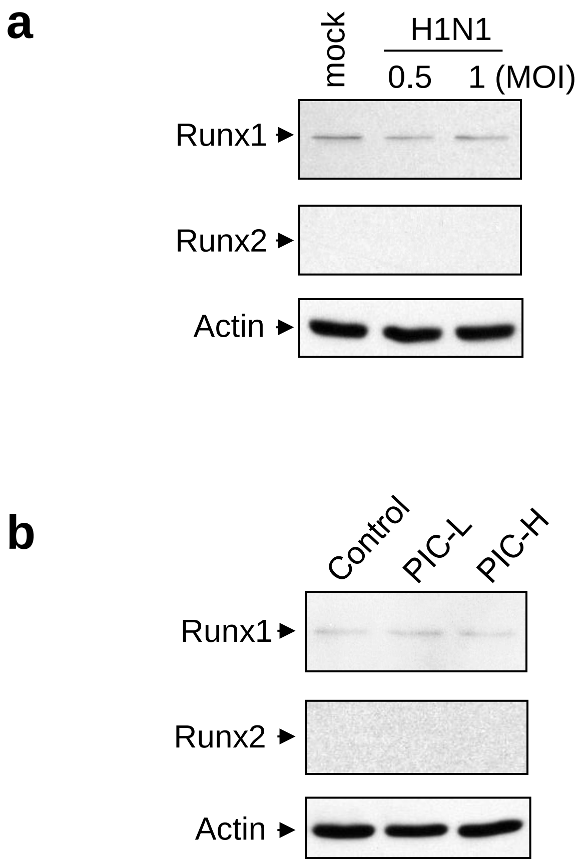

Supplementary Figure S1. IAV and dsRNA poly(I:C) do not induce the expression of Runx1 and Runx2. (a) BEAS-2B cells were treated with control PBS (mock) or infected with IAV H1N1 PR/8/34 strain at MOI of 0.5 and 1 for 24 h. (b) BEAS-2B cells were treated 24 h with control endotoxin-free PBS, low molecular weight poly(I:C) (PIC-L, 10 μg/ml) or high molecular weight poly(I:C) (PIC-H, 10 μg/ml). Equal amounts of cell lysates were subjected to Western blot analysis with specific antibodies against Runx1or Runx2.

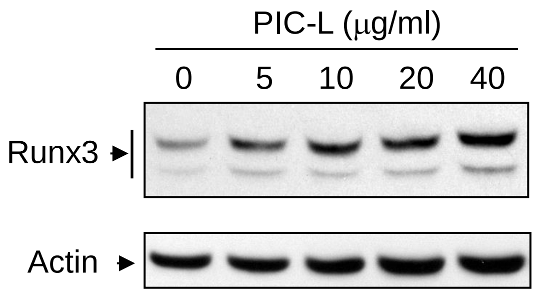

Supplementary Figure S2. Runx3 is induced by dsRNA poly(I:C) in 16HBE14o- airway epithelial cells. 16HBE14o- cells were treated 24 h with different doses of low molecular weight poly(I:C) (PIC-L). Cell lysates at equal protein amounts were subjected to Western blotting with Runx3 or actin antibodies.

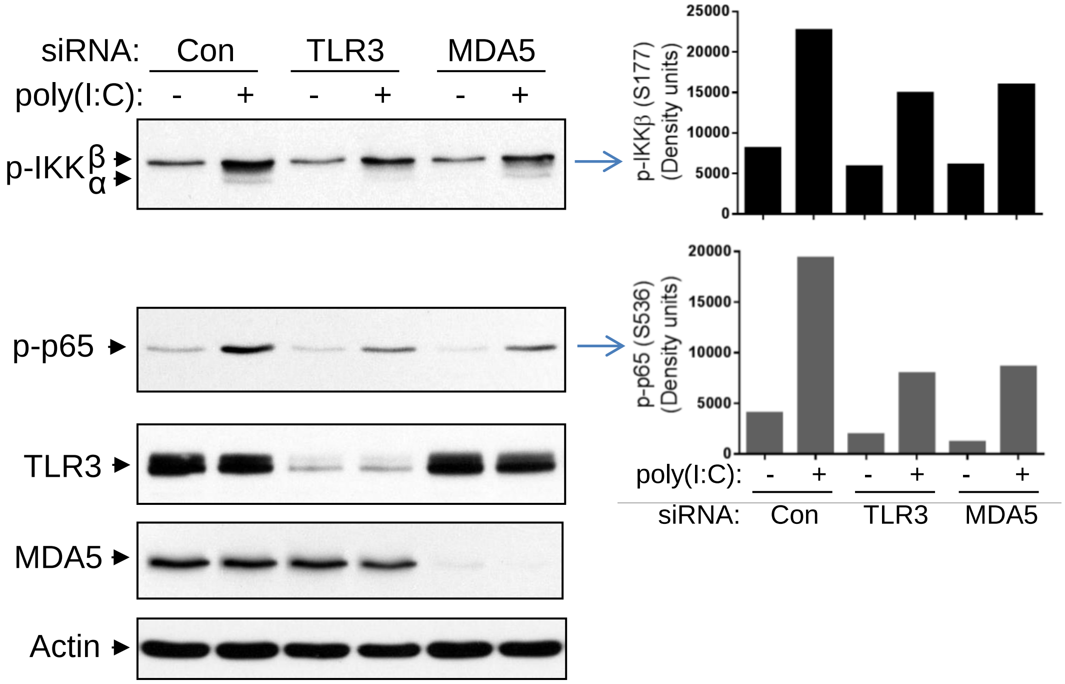

Supplementary Figure S3. TLR3 and MDA5 mediate poly(I:C)-induced phosphorylation of IKKα/β and NF-κB p65 in airway epithelial cells. BEAS-2B cells were transfected with 20 nM non-targeting control siRNA (Con), human TLR3 or MDA5 siRNAs, grown for 72 h then treated without (-) or with high molecular weight poly(I:C) (1 μg/ml) for 1 h. Equal amounts of cell lysates were subjected to Western blot analysis with phospho-IKKα (Ser176)/IKKβ (Ser177), phospho-p65 (Ser536), TLR3, MDA5 or actin antibodies. Results represent Western blots of two independent experiments.

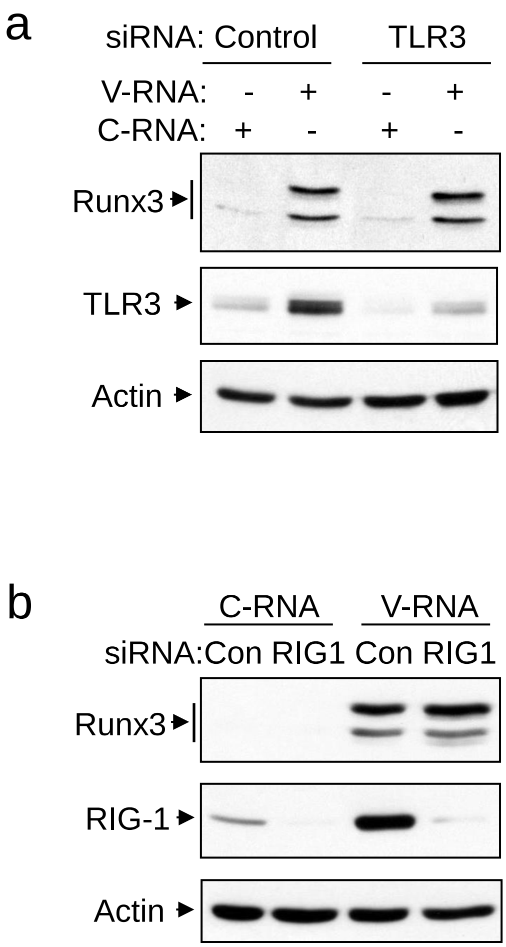

Supplementary Figure S4. TLR3 and RIG-1 are not involved in Runx3 induction by viral RNA. BEAS-2B cells were transfected with 20 nM non-targeting control siRNA (Con. Or Control), human TLR3 or RIG-1 siRNAs, grown for 72 h, then transfected with total RNA isolated from uninfected control BEAS-2B cells (C-RNA, 0.5 µg) or H1N1-infected BEAS-2B cells (V-RNA, 0.5 µg) for 24 h. Equal amounts of cell lysates were subjected to Western blotting with indicated antibodies. Results represent three independent experiments.

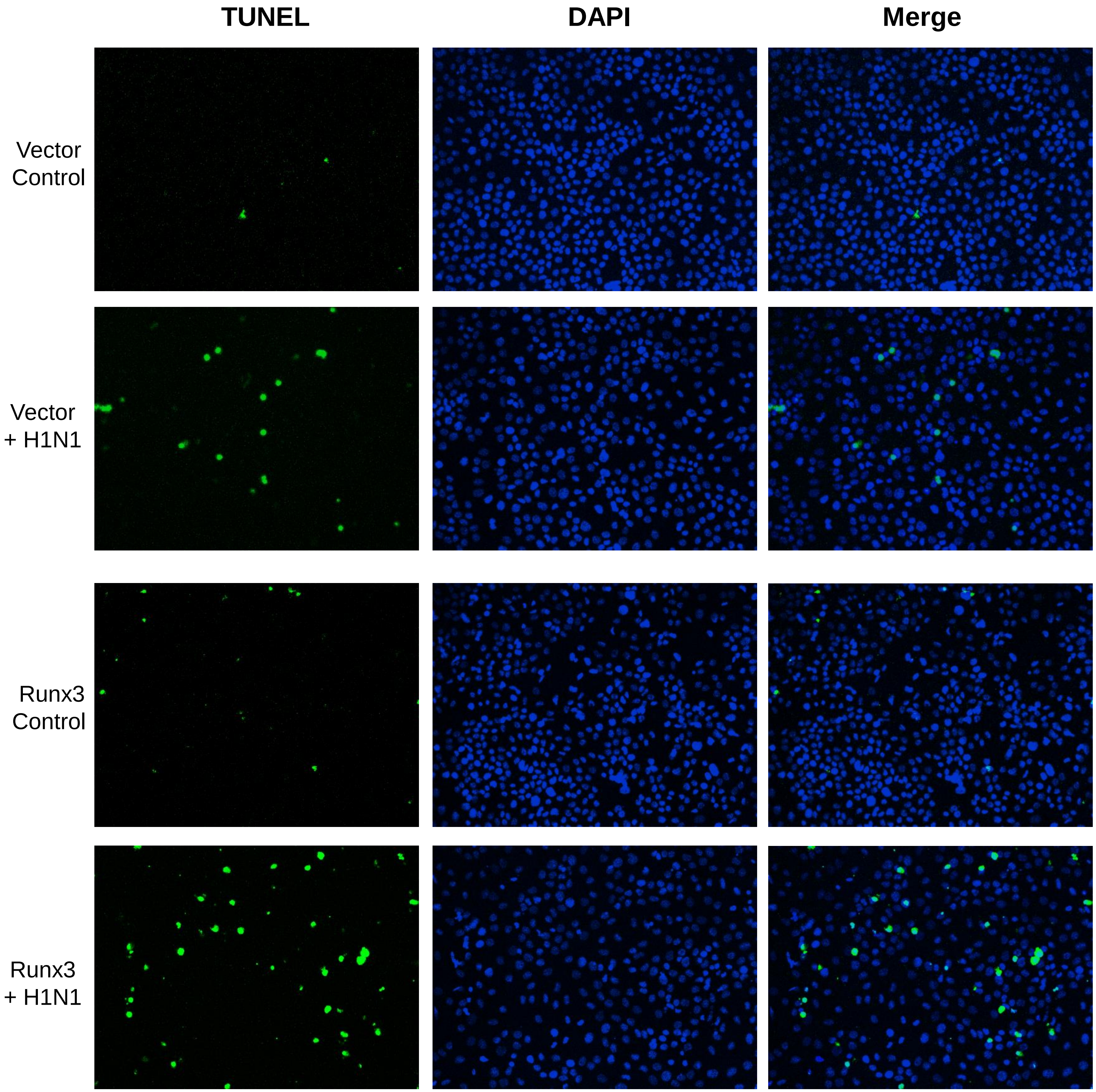

Supplementary Figure S5. Runx3 promotes airway epithelial cell apoptosis induced by IAV infection. BEAS-2B cells were infected with recombinant adenovirus containing vector alone or Runx3, grown for 60 h, then infected with (+) H1N1 PR/8/34 strain at MOI of 1 or treated with control PBS (Control) for 24 h. Cells were fixed and incubated with TUNEL assay reaction mixture containing FITC-12-dUTP or stained with DAPI as indicated. Fluorescence was visualized and images (final magnification: ×200) were captured by fluorescence microscopy. Results shown are representative images of three independent experiments.

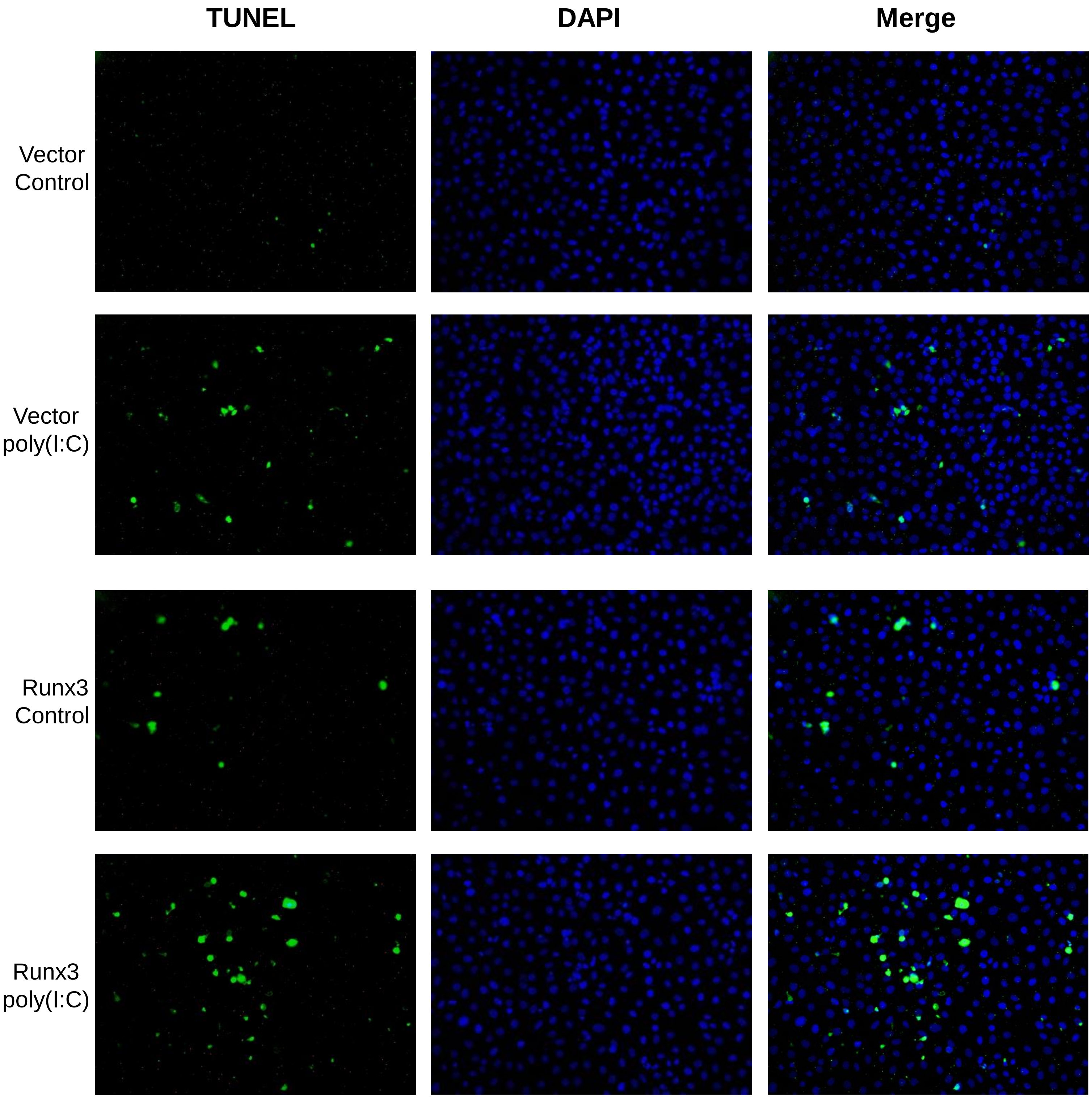

Supplementary Figure S6. Runx3 promotes airway epithelial cell apoptosis induced by dsRNA poly(I:C). BEAS-2B cells were infected with recombinant adenovirus containing vector alone or Runx3, grown for 60 h, then treated with control PBS (Control) or poly(I:C) (2 μg/ml) for 4 h. Cells were fixed and incubated with TUNEL assay reaction mixture containing FITC-12-dUTP or stained with DAPI as indicated. Fluorescence was visualized and images (final magnification: ×200) were captured by fluorescence microscopy. Results shown are representative images of three independent experiments.
